# eHealth policy framework in Low and Lower Middle-Income Countries; a PRISMA systematic review and analysis

**DOI:** 10.1186/s12913-023-09325-7

**Published:** 2023-04-01

**Authors:** Shegaw Anagaw Mengiste, Konstantinos Antypas, Marius Rohde Johannessen, Jörn Klein, Gholamhossein Kazemi

**Affiliations:** 1 Department of Business, History and Social Sciences, University of South-Eastern Norway, Vestfold, Vestfold Norway; 2 Department of Health Research, SINTEF Digital, Stiftelsen for Industriell Og Teknisk Forskning (SINTEF), Oslo, Oslo Norway; 3 Department of Nursing and Health Sciences, University of South-Eastern Norway, Porsgrunn, Norway

**Keywords:** eHealth policy framework, Developing countries, Low and Lower-Middle Income Countries, PRISMA systematic review

## Abstract

**Background:**

Low and lower middle-income countries suffer lack of healthcare providers and proper workforce education programs, a greater spread of illnesses, poor surveillance, efficient management, etc., which are addressable by a central policy framework implementation. Accordingly, an eHealth policy framework is required specifically for these countries to successfully implement eHealth solutions. This study explores existing frameworks and fills the gap by proposing an eHealth policy framework in the context of developing countries.

**Methods:**

This PRISMA-based (PRISMA Preferred Reporting Items For Systematic Reviews and Meta-Analyses) systematic review used Google Scholar, IEEE, Web of Science, and PubMed latest on 23^rd^ May 2022, explored 83 publications regarding eHealth policy frameworks, and extracted 11 publications scrutinizing eHealth policy frameworks in their title, abstract, or keywords. These publications were analyzed by using both expert opinion and Rstudio programming tools. They were explored based on their developing/developed countries’ context, research approach, main contribution, constructs/dimensions of the framework, and related categories. In addition, by using cloudword and latent semantic space techniques, the most discussed concepts and targeted keywords were explored and a correlation test was conducted to depict the important concepts mentioned in the related literature and extract their relation with the targeted keywords in the interest of this study.

**Results:**

Most of these publications do not develop or synthesize new frameworks for eHealth policy implementation, but rather introduce eHealth implementation frameworks, explain policy dimensions, identify and extract relevant components of existing frameworks or point out legal or other relevant eHealth implementation issues.

**Conclusion:**

After a thorough exploration of related literature, this study identified the main factors affecting an effective eHealth policy framework, found a gap in the context of developing countries, and proposed a four-step eHealth policy implementation guideline for successful implementation of eHealth in the context of developing. The limitation of this study is the lack of a proper amount of practically implemented eHealth policy framework cases in developing countries published in the literature for the review. Ultimately, this study is part of the BETTEReHEALTH (More information about the BETTEReHEALTH project at https://betterehealth.eu) project funded by the European Union Horizon’s 2020 under agreement number 101017450.

## Background

### eHealth in developing countries

eHealth is considered by many as a broad term containing a wide variety of digital health subdomains ranging from mobile health (mHealth) apps, electronic health records (EHRs), electronic medical records (EMRs), wearable devices, telehealth, and telemedicine, as well as personalized medicine [1–5]. As such, eHealth is defined and described by different scholars and institutions in different ways. For example, the World Health Organization (WHO) defined eHealth as “the cost-effective and secure use of Information and Communications Technologies (ICTs) in support of health and health-related fields, including health-care services, health surveillance, health literature, and health education, knowledge and research” [6]. Barbarella et al. [7] defined eHealth as an umbrella term that covers a wide range of health and care services delivered through ICTs such as EHRs, health management information systems (HMIS), telehealth, telemedicine, telecare, as well as tools for self-management, and health data analytics [7].

There is a big difference between the eHealth situation in developed and developing countries and this digital fraction is becoming a digital split [8]. Challenges of implementing eHealth differ greatly across nations as Upper Middle-Income Countries (UMICs) or developed countries are focusing on patient mobility and interoperability while Low and Lower Middle-Income Countries (LLMICs) encounter issues like lack of healthcare providers, problems of access to healthcare services, lack of proper education opportunities for healthcare providers, greater spread of illnesses, poor surveillance, and unconfident data management [8]. In most LLMICs, poor or vague eHealth strategy is a significant barrier to effective investment and implementation of sustainable eHealth solutions and establishing an eHealth favorable policy environment [9].

Despite continuous efforts toward health improvement in LLMICs, progress has been hindered by weak or dysfunctional health systems that fail to deliver qualified and affordable health care to populations in need [10]. LLMICs struggle with health system challenges related to weak governance, health workforce shortages, lack of legal and policy frameworks, and geographic and economic barriers to healthcare [11]. These challenges impede the effective delivery of health services to those in need. The rapid development of information and communication technologies (ICTs) over the last few decades offers new opportunities to address some of these challenges with innovative solutions. The delivery of health services using digital technologies in the context of LLMICs has been steadily growing in importance on the international public health policy agenda in the last 15 years [11]. Due to this, an increasing number of LLMICs are investing hugely in developing their technological infrastructure with appropriate national eHealth reform strategies aiming at improving quality and access to health services, while fostering transparency and governance [11].

### eHealth policy framework

Next to cost factors, lack of appropriate eHealth policy is the most mentioned factor that affects the successful implementation of eHealth strategy in most LLMICs [12]. eHealth policy-making is a complex process supposed to address several factors in a holistic manner to provide better health through better eHealth [13]. Theoretical approaches and existing evidence builds on the assumption that successful eHealth policy-making depends on four elements namely 1-Utilization of existing resources and evidence; 2-Addressing human factors; 3-Addressing technical factors; and 4-Addressing public policy factors [12–23].

Lack of adequate legislation and policy affects eHealth implementation both at the organizational and professional levels. Proper policies minimize data safety concerns, maximize liability, and simplify interoperable Electronic Health Record (HER) standardized exchange while maintaining integrity [17]. Incentives from the governments and stakeholders contribute to a smooth policy adoption path for healthcare providers by covering adoption costs in advance, providing implementation funds, and reimbursing in case of good performance [17]. Although eHealth is changing over time, standardization, interoperability, and policies are constant factors that influence it and are required to match these changes [17].

In this regard, WHO clarifies that a successful implementation of national eHealth includes a framework of strategic plans and policies in favor of healthcare development. These policies and strategies must protect citizens, bring equity, monitor cyber activities, ensure intersystem operability, and provide access to eHealth solutions for all citizens. Accordingly, the best approach to the implementation of such policies is a “glocal” approach that balances the experience and intelligence of global actors in eHealth adoption and implementation as well as the knowledge, expertise, and experience of local eHealth actors [8].

Generally, a framework is either a standalone or combination of a set of principles that guide research and development, a set of strategies that assist the development process, or a set of required constructs for quality enhancement [24]. Policy frameworks define the main actors and coordinate mechanisms to tackle the conflict of interests between the main players of an eHealth system while policies define priorities within a guiding framework in which stakeholders cooperate [25, 26]. EHealth policy is defined as “a set of statements, directives, regulations, laws, and judicial interpretations that direct and manage the life cycle of eHealth” [27]. Success in this context is measured by the greatness of addressing policy issues with jurisdictions, and failure can be the consequence of isolated decision-making [27]. EHealth policies can stand alone or be part of a bigger strategy or policy structure which are driven by the needs and activities of e-governments, standard organizations, industrial groups, academic institutions, and health service providers [8]. Although there is no clear formula, all of them require the same elements of cross-sectional technical and communicational infrastructure, interoperability, and user-centricity [28].

### eHealth policy framework in LLMICs

In recent years, many LLMICs are making efforts to develop a national eHealth policy and strategy to improve the quality and availability of health service delivery using digital health technologies and platforms. However, evidence showed that efforts are fragmented and the impact of existing policy frameworks on health service delivery is not explored properly [12].

In LLMICs, most of the eHealth organizations are segregated unconnected islands that are required to integrate with each other and take a need-driven approach to avoid failures resulting from the technology-pushed approach [18]. To make it happen, LLMICs must take into account basic administrative and health service processes, digitalization of basic administrative processes, basic Electronic Health Record (EHR) systems, surveillance and public health data collection, national framework platforms with common components for all the districts, and eHealth capacity development [18]. This can be a starting point that can be enhanced step by step by starting from regions populated between 5 to 10 million [18]. Moreover, such an implementation requires professional change management to diminish user resistance and guide the health providers to a less paper-based health service focusing on quality, gap identification, and need extraction [18]. Ultimately, policies should be fortified and supported by legislation and emphasize Transparency, Accountability, Participation, Integrity, and Capacity as suggested by the TAPIC framework introduced by WHO [18]. It is easy to copy and paste policies and strategies from developing countries; however, approaches must match local conditions and contextual factors with lessons learned from experienced countries. For instance, political and economic stability, high-level national leadership, full engagement of critical actors, long-term affordability and financial support, minimum ICT and eHealth capacity, and local ownership should be taken into account well before policy designation and implementation [18].

A 2018 study has explored Sub-Saharan African countries’ eHealth regulations readiness based on the aforementioned criteria and ranked them as the following; 1- Mauritius, 2- Botswana, 3- Seychelles, 4- Cape Verde, 5- Ghana, 6- Senegal, 7- Rwanda, 8- Namibia, 9- Uganda, Kenya, 10- Zimbabwe, 11- Gabon, 12- Mali, 13- Mozambique, 14- Nigeria, 15- Sudan, and 16- Zambia [18]. After a deep exploration, it is pointed out that an interoperable integrated eHealth implementation in LLMICs is hindered by varied health system tools in use, lack of a holistic integrated infrastructure, inadequate governance, and inability to enforce a greatly diverse group of health facility owners, donors, and financing resources, though the legislation does exist [18]. In order to create a contextualized effective eHealth policy framework, this study analyzes the literature regarding this topic as explained in the next section.

Accordingly, this study conducted a PRISMA-based systematic literature review on the current landscape of eHealth policy frameworks in the context of LLMICs and proposed a framework suitable for the development of eHealth policy in this context. In this regard, after exploring the current eHealth policy framework literature in the background section, the rest of this study includes, explaining the review process and analysis in the methods section, revealing findings in the result section, explaining findings in the discussion section, and pointing out implications/ in the conclusion section.

## Methods

This study uses the PRISMA method for systematic literature review and benefits expert opinions and tools of R programming for the analysis of the content. Moreover, the protocol of this systematic review has not been pre-registered in any of the renowned registries.

### Search process

This study is a review of eHealth policy frameworks in LLMICs and developing countries that use a systematic approach for the search process of prominent scholarly articles based on the guidelines introduced for writing systematic literature reviews and PRISMA methodology [29]. The review process started on 23^rd^ May 2022 with defining high-ranked search engines suitable for the topic of interest. Accordingly, Google Scholar, IEEE, Web of Science, and PubMed were selected to cover policy framework and health fields sufficiently. Due to the topic and research question, keywords were identified as “eHealth”, “e-health”, “digital health”, “telehealth”, “m-health”, “mhealth”, “telemedicine”, “policy”, “framework”, “low income”, “low and middle income” and “developing countries”. The whole process consists of 16 times of search queries including different combinations of keywords with the operators of “AND” and “OR” in the aforementioned search engines. Search strings on different search engines are listed in Table 1.

**Table 1:** Search Strings

Engine	Search String	Year Filter
Google Scholar	[["ehealth" OR "e-health" OR "digital health" OR "telemedicine" OR "telehealth" OR "m-health" OR "mhealth"] AND "policy" AND "framework"]	No filter
	[["ehealth" OR "e-health" OR "digital health" OR "telemedicine" OR "telehealth" OR "m-health" OR "mhealth"] AND "policy" AND "framework"]	2007–2022
	[[["ehealth" OR "e-health" OR "digital health" OR "telemedicine" OR "telehealth" OR "m-health" OR "mhealth"] AND "policy" AND "framework"] AND ["low income" OR "low and middle income" OR "developing countries"]]	No filter
	[[["ehealth" OR "e-health" OR "digital health" OR "telemedicine" OR "telehealth" OR "m-health" OR "mhealth"] AND "policy" AND "framework"] AND ["low income" OR "low and middle income" OR "developing countries"]]	2007—2022
IEEE	(("ehealth" OR "e-health" OR "digital health" OR "telemedicine" OR "telehealth" OR "m-health" OR "mhealth") AND "policy" AND "framework")	No filter
	(("ehealth" OR "e-health" OR "digital health" OR "telemedicine" OR "telehealth" OR "m-health" OR "mhealth") AND "policy" AND "framework")	2007–2022
	((("ehealth" OR "e-health" OR "digital health" OR "telemedicine" OR "telehealth" OR "m-health" OR "mhealth") AND "policy" AND "framework") AND ("low income" OR "low and middle income" OR "developing countries"))	No filter
	((("ehealth" OR "e-health" OR "digital health" OR "telemedicine" OR "telehealth" OR "m-health" OR "mhealth") AND "policy" AND "framework") AND ("low income" OR "low and middle income" OR "developing countries"))	2007–2022
Web of Science	(("ehealth" OR "e-health" OR "digital health" OR "telemedicine" OR "telehealth" OR "m-health" OR "mhealth") AND "policy" AND "framework")	No filter
	(("ehealth" OR "e-health" OR "digital health" OR "telemedicine" OR "telehealth" OR "m-health" OR "mhealth") AND "policy" AND "framework")	2007–2022
	((("ehealth" OR "e-health" OR "digital health" OR "telemedicine" OR "telehealth" OR "m-health" OR "mhealth") AND "policy" AND "framework") AND ("low income" OR "low and middle income" OR "developing countries"))	No filter
	((("ehealth" OR "e-health" OR "digital health" OR "telemedicine" OR "telehealth" OR "m-health" OR "mhealth") AND "policy" AND "framework") AND ("low income" OR "low and middle income" OR "developing countries"))	2007–2022
PubMed	(("ehealth" OR "e-health" OR "digital health" OR "telemedicine" OR "telehealth" OR "m-health" OR "mhealth") AND "policy" AND "framework")	No filter
	(("ehealth" OR "e-health" OR "digital health" OR "telemedicine" OR "telehealth" OR "m-health" OR "mhealth") AND "policy" AND "framework")	2007–2022
	((("ehealth" OR "e-health" OR "digital health" OR "telemedicine" OR "telehealth" OR "m-health" OR "mhealth") AND "policy" AND "framework") AND ("low income" OR "low and middle income" OR "developing countries"))	No filter
	((("ehealth" OR "e-health" OR "digital health" OR "telemedicine" OR "telehealth" OR "m-health" OR "mhealth") AND "policy" AND "framework") AND ("low income" OR "low and middle income" OR "developing countries"))	2007–2022

To extract grounding and foundational articles around the topic, the queries started by including keywords of eHealth, policy, framework, low income, middle income, and developing countries with no filter for the publication year. However, citation of the articles based on the publication year and authority of the journals based on the H5 index were considered as the main factors of selection. Queries were then filtered by the publication year and publications from 2007 to 2022 were investigated separately. This filtration helps distinguish the grounding articles from top recent and state-of-the-art articles around the topic. Finally, after three rounds of filtration, 83 articles and proceedings were eligible based on the considered inclusion and exclusion criteria. Including publications have either explained, reviewed, proposed, or developed eHealth policy or policy frameworks in their text. Only publications published in the English language were considered for review. 4 Experts, Independently and also through meetings studied abstracts and conclusions to reduce the risk of bias and extracted specifically 11 publications scrutinizing eHealth policy frameworks. The first round of filtration started with reading the title of the article to see if it is related at all, and in the second round, the publication year, citation, and authority of the article were taken into account to increase the validity and authority. Ultimately, the abstract and conclusion of the articles from the second round were read to indicate the relevance and eligibility of the publication.

**Fig. 1:**
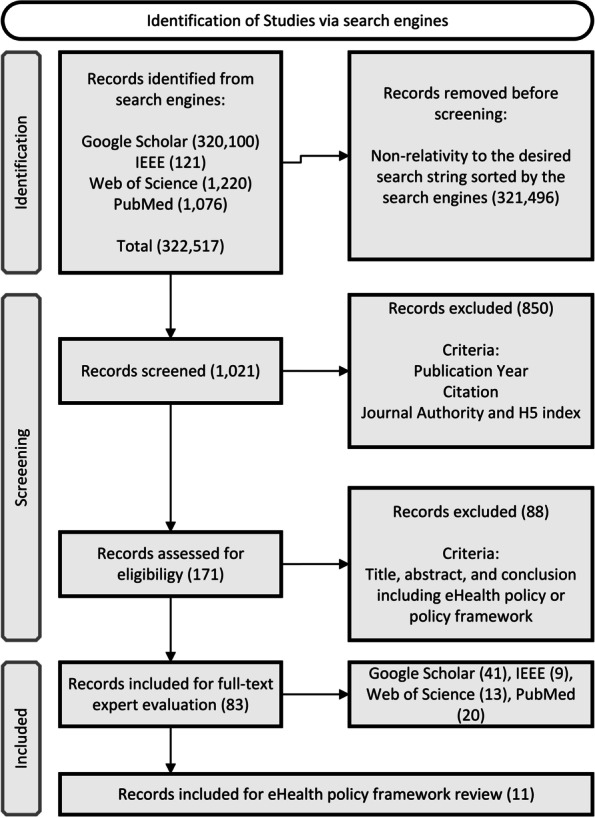
Search Process Flow Diagram

The average citation of all the articles is 59.5 which is at an acceptable level, and the average citation for the articles from 2007 up to now is 60.15 holding 96% of the portion that indicates the significance of the publications in this period. It is crystal clear that research around the combination of eHealth, policy framework, and LLMICs and developing countries is increasing greatly due to the fact that 96% of the publications belong to the last 15 years. Although, only a very few numbers of them are concentrating on LLMICs and developing countries. A flow diagram of the whole search process is illustrated in Fig. 1.

### Analysis process

The analysis was conducted by reading the papers and assessing: 1) is the research explicitly placed in an LLMIC context? 2) research approach used in the paper: literature review or empirical? If empriical – what kind of data and methodology. 3) The main contribution, as stated by the authors 4) constructs/themes/dimensions used by the framework mentioned in the paper, and 5) Relation to categories used by the BettereHealth project (see Table 4). The main purpose of this analysis was exploration of how existing frameworks relate to the overall objectives of the BettereHealth project.

Of these, numbers 4 (contents of framework) and 5 (categorization according to BettereHealth project) can be open for bias and interpretation. In order to minimize the risk and ensure that interpretation was in line with the overall project, one of the authors conducted the initial analysis, after which the remaining authors read the papers and made their own interpretations. Finally, the authors held a workshop where we discussed and agreed upon the structure and interpretations found in this paper.

To have a better-detailed view of the eHealth policy framework in LLMIC, publications that have explicitly mentioned the word “framework” in their title or keywords were identified and their main contribution, research approach, and framework dimensions or constructions were extracted. As a result, 11 publications were extracted and only 4 of them mentioned LLMIC in their contribution. 5 publications were literature or desk reviews, 3 were including conceptual frameworks, 2 were including expert interviews, and 1 included a content analysis regarding eHealth policy frameworks.

In addition, to extract the most discussed concepts from the selected publications and investigate the frequency of desired concepts in their text, the RStudio programming tool was used to collect publications and conduct a word frequency analysis with the help of the Natural Language Processing packages. For data collection and data mining, Rstudio was used due to its capabilities in the extraction of concepts and language processing analysis [30]. In this regard, two methods were mixed; first, top-quality publications were extracted, as explained in Sect. 3.1., the first step provided 83 documents. Second, the Rstudio programming tool was used to grab and mine the data from the 83 pdf documents. All the collected publications, conducted programming procedures, and analysis results are stored offline in the possession of the BETTEReHEALTH project.

To collect and clean the data, the packages and libraries of writexl, wordcloud, wordcloud2, tm, lsa, nlp, corpus, readr, devtools, stringr, etc. were used along with some functions like tm_map, removewords, stopwords, stemdocumen, stemcompletion, termdocumentmatrix, documenttermmatrix, removepunctuation, removenumbers, stripwhitespace, etc. [30, 31]. This way it is possible to conduct a thorough document analysis via Natural Language Processing tools that explore word and concept frequency and correlational relationships between a set of documents [30, 31].

Ultimately, different visualization and exporting methods were used to export the analyzed data from Rstudio [30]. For this purpose, Text Mining, Latent Semantic Analysis, and wordcloud2 were used to extract the most frequent concepts and words from the pdf texts [31, 32]. The Latent Semantic Analysis (LSA) creates a well-connected representation of the publications. LSA creates a latent semantic space of concepts and words from publications, and then links these spaces and extracts the coherence of the concepts in them [31, 33].

## Results

Due to the chosen analytical methodology, the findings are separated into two sections of expert analysis including expert reviews of the chosen articles, and R programming analysis including the extraction of the most discussed concepts and document-term-matrix correlation based on the Pearson method.

### Expert analysis

Exploring eHealth policy frameworks in LLMICs, a summary of 11 of the publications identified in our literature review mentioning "framework" in their title, abstract, conclusion, or keywords is presented in the next paragraphs. Here, we present a summary of the LLMICs context, main contributions, research approach, and framework dimensions/constructs in these articles.

Gemert-Pijnen et al. 2011 [24] is a literature review study presenting no LLMICs context. This study contributes to creating a holistic eHealth “Uptake and Improvement” framework by adding the User Experience (UX) component to it, based on 16 reviewed frameworks. They introduce 6 working principles as the following; 1- Stakeholder participation through the whole development process, 2- Continuous cyclic longitudinal evaluation through the whole development process, 3- Consideration of implementation and its issues from contextualization and value specification until design and operationalization, 4- Organizational process and infrastructure reshape, 5- Persuasive and bonding technology-user relationship, and 6- Advanced impact assessment. They refer to user-centricity, interoperability, and policy implementation [24].

Khumalo 2017 [25] is a literature review study presenting an LLMIC context. This study contributes to recommending eHealth legislation implementations in Zimbabwe. They emphasize legislation to control the use of technology systems, and highlight integrity, accessibility, efficacy, and security of data, and evidence-based record management along with the necessity of upholding health ICT laws and interoperability standards at a national and international level. They point out the requirements as the following; 1- Public and private sector uniformity, 2- Cross-sector information sharing, 3- Regulations for national healthcare integration, 4- Fortification of policies by legislation, 5- Ensuring data availability, access, confidentiality, accountability, interoperability, exchange, quality, and sharing through legislation, 6- Ethical consideration and actor identification through legislation. They refer to legal issues of policy implementation [25].

Mburu & Kamau 2018 [34] is a PRISMA systematic review study presenting an LLMIC context. This study contributes to introducing a three-component eHealth implementation framework for Kenya considering socioeconomic and technical challenges such as cultural barriers, inadequate funding, changing priorities, political uncertainties, inadequate technical skills, limited health information sharing, undue influence of developers, and change resistance. They propose a three-component framework including governance, guiding principles, and predictable policy development. In this regard, governance emphasizes leadership, oversight, and administrative support for eHealth policy development and implementation, guiding principles include best global-consistent ideologies and values supporting vision, mission, values, priorities, legislation, and governing, and finally, predictable policy development process includes structured policy development by needs assessment, planning and design, policy drafting, draft validation, policy approval, policy implementation, and review and evaluation. They refer to human empowerment and policy roadmap [34].

Katehakis & Kourabali 2019 [35] propose a conceptual framework with no LLMIC context. This study contributes to the creation of an interoperability management framework based on the EU context prone to patient empowerment and emphasis on sustainability, legislation of framework and governance, and technology maturity. They propose a four-layered interoperability framework including legal, organizational, semantic, and technical layers based on 12 principles for interoperability based on the EU framework as the following; subsidiary and proportionality, openness, transparency, reusability, technological neutrality and data portability, user-centricity, inclusion and accessibility, security and privacy, multilingualism, administrative simplification, preservation of information, and assessment of effectiveness. They refer to technical interoperability and user centricity [35].

Scott & Mars 2013 [9] is a study proposing a strategy development framework based on conceptual frameworks, strategy theories, and complex system analysis with no LLMIC context. This study contributes to the introduction of 7 principles for eHealth strategy development, the creation of an 8-step interoperability management framework, and the differentiation of strategies and policies. Principles are the simplification of complex contexts, acquisition of a pragmatic approach, dispersion of costs, application of balanced eHealth components, appropriation of eHealth solutions and settings, and provision of long-, and medium-term targeting. They highlight that strategies indicate “Where” and “Why” while policies indicate “How” regarding the actions. Ultimately, they create the steps as the following; 1- Evidence gathering and situation assessment at the institution, country, and international level, 2- holistic review of poverty, economic policy framework, physical geography, governance issues and political stability, cultural barriers, geopolitics, resource issues, eHealth readiness, linkages, infrastructure, and infostructure, 3- Differential diagnosis based on the information and analysis from step 1 and 2, 4- Preliminary prioritization based on disease burden, determinants, knowledge, economic costs, and resources, 5- Identifying solutions by using material and analysis from steps 1 to 4, 6- Considering eHealth solutions with a focus on top 20%, 7- Secondary prioritization based on potential costs, the complexity of implementation, implementation readiness, and population proportion, and 8- Strategy formulation at the institution, local, regional, and country-level based on the information and analysis from steps 1 to 7. They refer to policy roadmap implementation [9].

Mauco et al. 2019 [36] is an expert interview study with no LLMIC context. This study contributes to the proposition of an eHealth readiness assessment framework, identification of 7 main actors of eHealth implementation, and mismatch extraction between expert reviews and literatures’ eHealth readiness themes. They identify the actors as communities, government, private sector, state-owned enterprises, statutory cooperations, international agencies, and international partnerships. Moreover, they reflect 4 expert opinions and 8 literature-based themes for eHealth readiness as the following. Expert opinions themes are governance, stakeholder issues, resources, and access, and literature-based themes are organizational, technological-infrastructural, government, societal, healthcare provider, engagement, core, and public-patient readiness. They refer to human empowerment and capacity building [36].

Mauco et al. 2020 [37] is an expert panel interview study with no LLMICs context. This study contributes to the validation of the previously proposed eHealth readiness assessment framework by evidence establishment and develops the previous framework by dividing it into the stakeholders-engagement and eHealth infostructure and infrastructure readiness assessment. In this regard, stakeholder engagement includes the government at the core and community leaders, Non-Governmental Organizations (NGOs), the private sector, and international partners, etc. as the supplementary factors. Also, infostructure/infrastructure includes governance, national eHealth strategy development process, and access to the core. Furthermore, they divide the governance layers to national and international layers with supplementary factors of societal, organizational, governmental, and technological/infrastructural readiness, and divide the national eHealth strategy development processes layers into the resources layer and stakeholder issues layer. The former includes technological/infrastructural readiness as a supplementary factor, and the latter includes core, healthcare provider, engagement, and public/patient readiness as supplementary factors. They refer to human empowerment, capacity building, and technical infrastructure [37].

Vis et al. 2020 [38] is a systematic review study with no LLMIC context. This study contributes to the identification of the multi-perspective and multi-impact assessment approach as a necessity for sustainable incorporation of eHealth in traditional healthcare, and extracts and compares 3 staged, 13 dimensional, 3 hybrid, and 2 business modeling frameworks for eHealth implementation. They identify dimensions extracted from the reviewed studies as the following; technical performance/functionality, cost, clinical outcomes, organizational aspects, system-level aspects, and outcomes and methods of assessment. They refer to infrastructural interoperability, policy-financial issues implementation, and human capacity building [38].

Andreeva et al. 2020 [39] is a conceptual study with no LLMIC context. This study contributes to highlighting typical European eHealth development trends for legal framework implementation, analyzes Bulgaria’s national health digitalization through eHealth functioning and adequacy, and compares EU and national eHealth strategy formulation. They indicate that the EU focuses on providing cross-boundary lifesaving information, policy inclusion and coordination between EU countries, and engagement of patients and professionals in the adoption and implementation of eHealth strategies, while Bulgaria focuses on national standards for health information and statistics, security and interoperability policies, the establishment of publicly accessible national health information systems, modular real-time cross-sectional health information exchange, providing connectivity of medical providers, secured centralized registry with authorized access, web-based service and encrypted data delivery, and design, refinement and implementation of eHealth development in a connected network. They refer to policy legal issues [39].

Kante & Ndayizigamiye 2021 [40] is a content analysis study with LLMIC context. This study contributes to the identification and categorization of actors as the core element, and context, content, and process as the angles of the eHealth policy triangle framework in South Africa. They also highlight the Internet of Medical Things (IoMT) as a potential eHealth game changer for elderlies in LLMICs. They introduce a four-dimension policy/strategy framework including actors, contexts, content, and process. In this regard actors' core includes government, universities, districts, provinces, the Health Professional Council of South Africa, the United States Agency for International Developments, Novartis Foundations, WHO, Telecommunication Union, African Center for eHealth Excellence, NGOs, and healthcare professionals. Also, the context angle includes situational, structural, cultural, and international factors, and the content angle includes the implementation of comprehensive EHRs, improvement of efficacy and quality of HR and medication access at the institutional level through digitalization, the establishment of interoperable systems, vulnerable group healthcare scale-up, and developing digital health workers’ knowledge. Finally, the process angle includes problem identification, legitimacy, and feasibility and evaluation. They refer to human capacity building, infrastructural interoperability, and policy implementation [40].

Semwagna et al. 2021 [41] is a literature review study with LLMICs context. This study contributes to the proposition of an 8-dimension eHealth adoption framework for developing countries like Uganda and its validation via a two-tier process with experts from academia and industry. They propose dimensions as the following; 1- Socio-Demographic dimension including characteristics of the community like age, gender, education, occupation, income, eHealth awareness, and mobile access, 2- Socio-Cultural dimension including social and cultural aspects of the community like beliefs, friends’ influence, power and masculinity, and distance to the nearest health facility, 3- Technology dimension including infrastructure and technology readiness, availability of organizational and technical infrastructure, presence of integrity and interoperability standards, development of IT agendas, and vendor support, 4- Information dimension including patient-friendly message exchange, high-quality information, and private and confidential information with authorized access, 5- Organizational dimension including environmental adequacy and readiness, consistency with organizational core missions, workforce ICT skills, appropriate information culture, addressing HER use resistance by voluntary participation, performance improvement, and knowledge strengthening, 6- Governance dimension including support from top level management, and design and implementation of governmental eHealth policies, 7- Ethical and Legal dimension including considerations regarding collection, process, and use of patients’ data, and 8- Finance dimension including adequate financing for implementation, operation, training, awareness, and maintenance. They refer to human capacity building, infrastructure interoperability, and policy implementation [41].

### R programming analysis

**Fig. 2:**
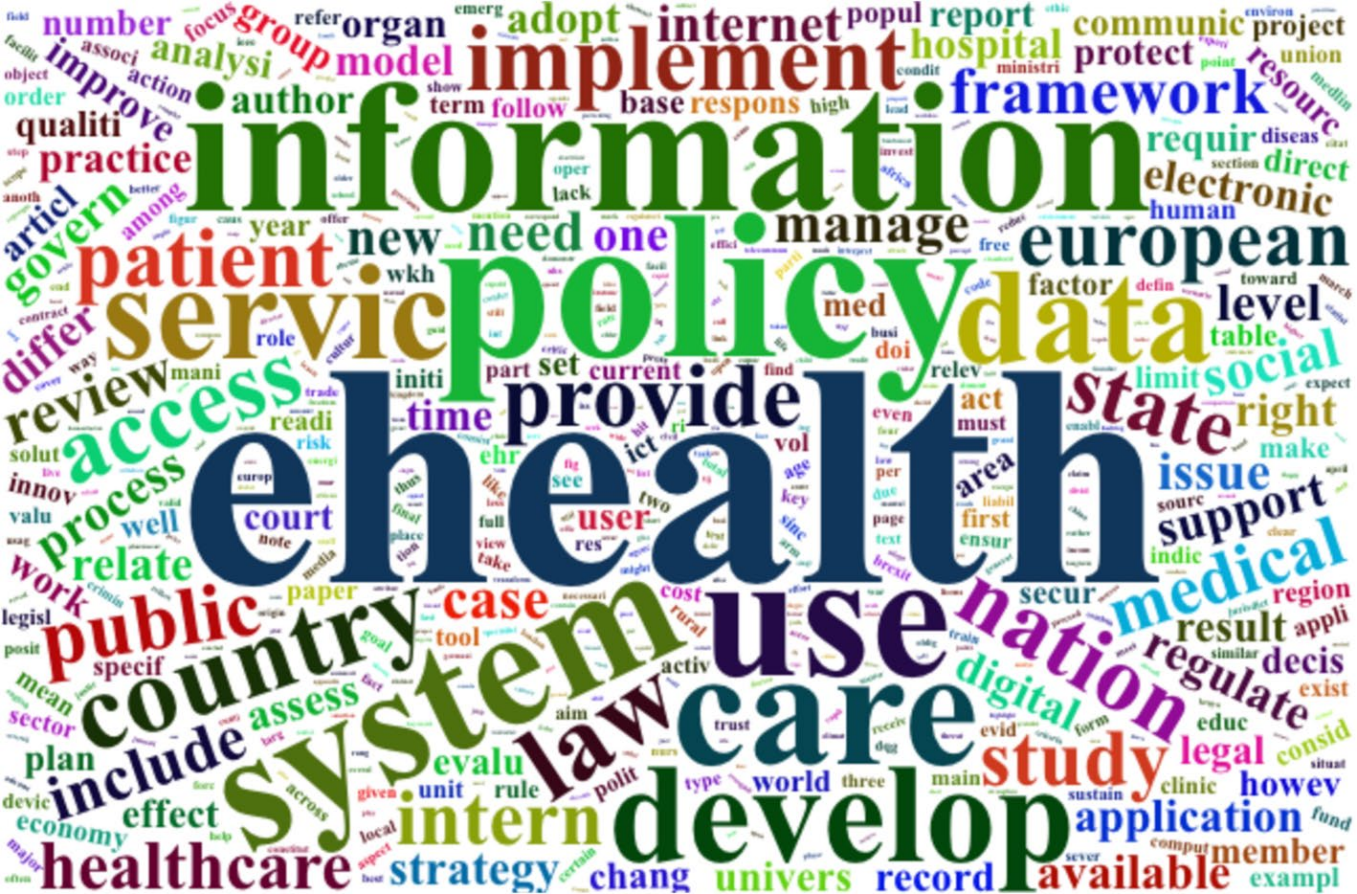
Cloud word of the most discussed concepts in the publications

Totally, 483,149 words were mined. A word frequency analysis conducted with the help of wordcloud2 in the RStudio environment is depicted in Fig. 2. After text collection, cleaning, preparation, and mining, a final manual check was conducted to remove non-essential words that were not filtered in the cleaning phase. Also, words with a frequency less than 100 were removed from the wordcloud.

Table 2 indicates the top 10 discussed concepts in the publications collected from Google Scholar, IEEE, Web of Science, and PubMed. Since this review is about ehealth policy framework in LMICs, it is crystal clear that these words will appear in the top 10. Accordingly, words like health, ehealth, framework, and policy were removed from the top 10 discussed concepts to have a better view of the discussed concepts.

**Table 2:** The top 10 discussed concepts in the publications

Concept	Information	Use	System	Develop	Service	Data	Technology	Country	Law	Access
Occurrence	3673	3499	3313	2750	2704	2661	2567	2282	2165	1907

Also, Table 3 indicates the occurrence of the indicated keywords; such as entrepreneurship, startup, patient-centered, user-centered, human-centered, perspective, involve, technical, technology, and capacity.

**Table 3:** Occurrence of the indicated keywords

Concept	Entrepreneurship	startup	Patient-centered	User-centered	Human-centered	perspective	involve	Technical + technology	capacity
Occurrence	38	12	66	4	5	3	414	3054	157
Concept	interoperability	trial	study	innovate	roadmap	research	Build	private	standard
Occurrence	291	129	8	561	60	1834	252	444	734
Concept	infrastructure	human	ehealth	framework	empower	universal	clinic	access	financial
Occurrence	440	680	6149	1291	52	3	482	1907	403
Concept	user friendly	user	policy	legal	strategy	standard	LLMIC	engage	implement
Occurrence	26	830	4108	836	1223	734	50	233	1900

With regards to the occurrence of the abovementioned keywords, it is extracted that in an eHealth context, policy is one of the most important factors followed by technology, access, implement, research, and standard. However, concepts like entrepreneurship, startup, user/patient/human-centered, roadmap, universal, empower, user-friendly, and LLMIC are not discussed enough and require further investigation.

**Fig. 3:**
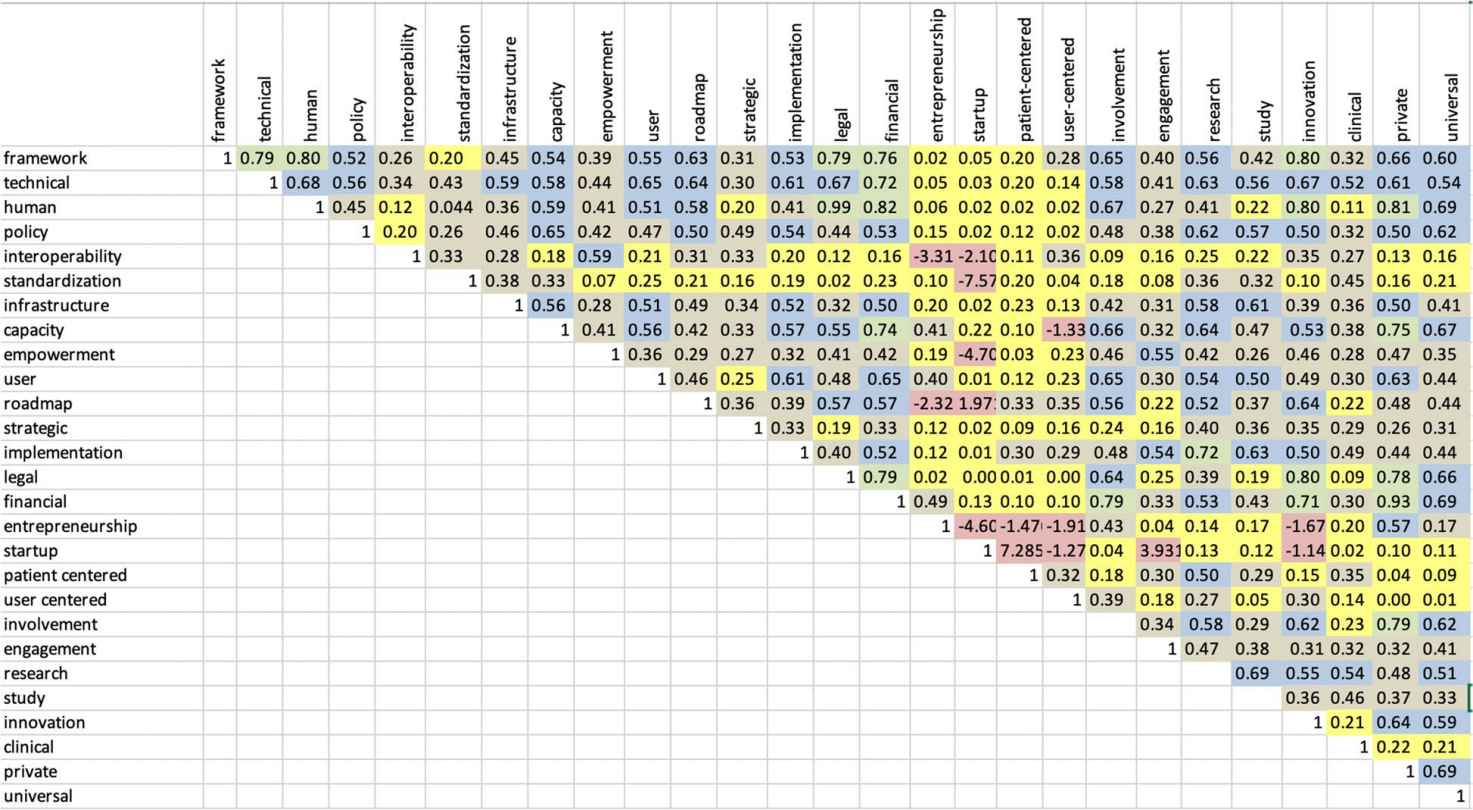
Between document correlation by keywords

The Latent Semantic Analysis (LSA) is used to create a well-connected representation of the publications. LSA creates a semantic space from the concepts extracted from the publications. It is a tool to measure the coherence of the text by comparing the vectors for two adjoining segments of text in a high-dimensional semantic space. It identifies the degree of semantic relatedness between the segments [31, 33]. With the help of LSA and Cosine and Cor functions, we were able to extract and observe how much do documents correlate to each other when they discuss different issues. For example, when documents discuss the framework and legal issues, their notions correlate by 0.79. Figure 3 indicates a between-document correlation based on different keywords using LSA.

For a better understanding, correlations are colored based on the below numbers.
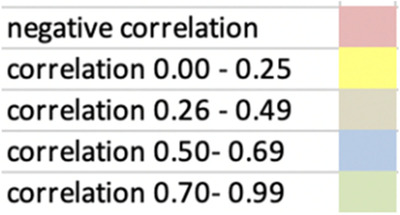


As it is clear from the correlation figure above, when documents discuss frameworks their notions correlate strongly with technical, human, legal, financial, and innovation, and correlates more than half with policy, capacity, user, roadmap, implementation, involvement, and research, and correlates less than half with interoperability, infrastructure, empowerment, strategy, user-centered, engagement, study, and clinical, and correlates less than 0.25 with standards, entrepreneurship, startup, and patient-centered. Here also, the importance of technical, human, and legal factors are pointed out in developing a framework while policy, roadmap, involvement, interoperability, empowerment, user-centricity, engagement, and standard concepts are not showing a well-deserved correlation with the notion of the studies discussing frameworks. In this regard, this gap should be addressed by developing an eHealth policy framework capable of providing interoperability and user-centricity for developing countries in order to address their challenges in implementing an effective eHealth solution.

## Discussion

Scrutinizing the main contribution of each study, it is clear that not all of them actually develop or synthesize new frameworks for eHealth policy implementation, but rather introduce eHealth implementation frameworks, explain policy dimensions, identify and extract relevant components of existing frameworks or point out legal or other relevant eHealth implementation issues. On one hand, most of the studies present new frameworks or frameworks synthesized from existing literature. On the other hand, none of them actually presents a framework for the implementation of eHealth policy. These frameworks each have a different aim. Some of them are directional and describe a process-based approach for eHealth implementation, eHealth development, eHealth impact assessment, and eHealth readiness assessment while others are more specialized and focus on interoperability management, strategy development, and legislation requirements. For instance, [24] is focusing on eHealth uptake and improvement by pointing out the importance of UX and introduces 8 pragmatic principles including stakeholder participation, continuous evaluation, implementation contextualization, organizational reshaping, user-bonding technology, and advanced impact assessment. Moreover, [35] introduces a framework that specifically addresses interoperability management prone to patient empowerment with emphasis on sustainability, legislation of framework and governance, and technology maturity. They also clarify 12 principles for interoperability management based on the EU’s framework. In another example, [36] presents a framework that assesses eHealth projects' readiness by identifying 7 main involved actors of eHealth implementations, actors are namely, communities, government, private sector, state-owned enterprises, statutory corporations, international agencies, and international partnerships. They have also compared experts’ opinions with eHealth literature regarding readiness themes and found mismatches that have led them to the development and validation of an eHealth readiness framework based on the mixture of the aforementioned themes.

[38] do not create a new framework, but rather examine existing frameworks and summarize the dimensions and types of frameworks presented in the existing literature. They extracted and compared 3 staged, 13 dimensional, 3 hybrid, and 2 business modeling frameworks for eHealth implementations and identified the multi-perspective and multi-method impact assessment approach as a necessity for sustainable incorporation of eHealth in traditional healthcare.

Regarding policy and legislation, only four of the publications have mentioned these concepts in their content. Although, with regards to the most discussed concepts in the RProgramming analysis section, the coexistence of the previously mentioned concepts in the most discussed concepts reveals how important is legislation (Law) for a better accessed (Access) and implemented (Develop) technology-based health data management system (Information, Data, System, Service, Technology).

For example, [25] recommends legislation and explains its requirements. This case recommends legislation for controlling the use of technology, data management, evidence gathering, and national and international health ICT implementation. In this case, uniformity of the stakeholders, cross-sectionality, national regulation, policy fortification, data management, interoperability, and ethical considerations are required to be addressed by legislation. Or in another example, [9] introduces 7 principles for eHealth strategy development through an 8-step plan. In this case, context simplification, pragmatic approach acquisition, cost dispersion, eHealth application balance, solution appropriation, and long and medium-term provisioning are pointed out to be addressed through evidence gathering, holistic governance, diagnosis, prioritization, solution identification, top 20% eHealth solution consideration, second prioritization, and strategy formulation. They also emphasize interoperability and differentiate strategy and policy. In their opinion, strategy refers to “Where” and “Why” while policy refers to “How” when taking an action. In the EU-based review of [39], they indicate that an EU-compatible legal framework implementation should consider cross-boundary vital information sharing, policy inclusion, cross-continent coordination, and patient and healthcare worker participation in the strategy formulation. The last example is [40] which introduces four dimensions for policy frameworks. Between these dimensions, actors are at the core, and context, content, and process are the angles. The actor dimension includes stakeholders like government, universities, districts, providers, professionals, and national and international stakeholders such as WHO. The content dimension includes comprehensive health data management, efficiency and quality of healthcare enhancement, and interoperability. The context dimension includes situational, structural, cultural, and international considerations. Ultimately, the process dimension includes problem identification, legitimacy, feasibility, and evaluation.

Only four of the publications explicitly stated that they have an LLMIC context, while the other seven publications either did not mention the context or were based on an EU context [35, 39]. These publications still point out important directions relevant to developing countries, but context needs to be added while developing policy frameworks for each individual country.

Moreover, as indicated in the RProgramming analysis section, considering the occurrence of targeted keywords, the gap of entrepreneurial activities, patient-centricity, roadmap, LLMIC, and universal design deliver the fact that developing countries require more attention regarding their eHealth implementation roadmaps, specifically with regards to the policy as it is the most discussed concept after eHealth in these publications. Moreover, private sector and user participation are also required to better implement such projects with a patient-centered approach. Finally, legal, human, infrastructure, interoperability, innovate, private, financial, and engage concepts are discussed, but not as much as their significance requires. Noteworthy that financial, interoperability, workforce, and private sector inclusion and consideration are vital for eHealth policy implementation.

In terms of the research approach, only two of the publications rely on empirical data [36, 37], four are literature reviews of other frameworks, one is a content analysis of strategy documents, and the remaining four are conceptual, with no mention of an empirical basis for construct development. Only one of the publications has explicitly validated their framework [37].

Finally, examining the contents of the frameworks reveals their deep differences. Some rely on a set of dimensions, or constructs [9, 34, 38, 40, 41] and some provide a set of principles, propositions, or pieces of advice [9, 24, 25, 39], while the third category of frameworks address broader themes such as governance or stakeholder issues [36, 40].

In the Expert Opinion section, we have extracted the contents of the different frameworks based on the categories used by the BettereHealth project. It is noteworthy that none of the frameworks explicitly address technical standardization issues. Though, the lack of standardization is an important issue for eHealth technologies and services. Similarly, only one framework explicitly includes user-friendliness and user-centeredness as a category. Though, user involvement and usability have been important parts of the literature for decades. On the other end of the scale, the technical category infrastructure is part of four publications, the human category of capacity building is addressed by five, and the policy categories of strategic implementation and legal/financial issues by four of the frameworks as indicated below in Table 4.

**Table 4:** Contents of different eHealth policy frameworks

Publication	Technical			Human			Policy		
	Interoperability	Standardization	infrastructure	Capacity building	Empowerment	User friendliness	Roadmap	Strategic implementation	Legal and financial
Gemert-Pijnen et al. 2011 [24]	X					X		X	
Khumalo 2017 [25]									X
Mburu & Kamau 2018 [34]					X		X		
Katehakis & Kourabali 2019 [35]	X								
Scott & Mars 2013 [9]							X	X	
Mauco, Scott, Mars 2019 [36]				X	X				
Mauco et al. 2020 [37]			X	X	X				
Vis et al. 2020 [38]	X		X	X				X	X
Andreeva, Yolova & Dimitrova 2020 [39]									X
Kante & Ndayizigamiye 2021 [40]			X	X				X	
Semwanga et al. 2021 [41]			X	X					X

Finally, while only four publications explicitly mention a roadmap or point out the dimensions or requirements for policy implementation, many of them mostly indicate some kind of process or direction, something which is also noted in the review of frameworks by [9, 25, 38–40]. We suggest that step-by-step process is an important aspect when creating a framework for policy implementation, and suggest at least four steps in this process, as below.

### Step 1- before policy creation

Our review reveals that stakeholder readiness and participation, process formulation, cross-boundary consideration, human factors (skills, knowledge, motivation) centricity, legislative fortification, financial prioritization, and contextual infrastructures are all critical when it comes to what kind of eHealth technology can be implemented. Thus, the first step should be to map these aspects.

### Step 2- develop policy based on realistic expectations

Further, our review revealed the importance of developing policy frameworks based on realistic expectations. More specifically, it points to the following important aspects of policy creation such as international, national, regional, local, and institutional situations, and geographical, economic, cultural, infrastructural, and resource-related issues that should be considered in the eHealth policy design and prioritization process. Policy development should be backed up by legislation and must have short-term and long-term provisions based on realistic cultural, situational, financial, national, and international situations. Moreover, contextualization is an important part of policy development due to the importance of health data standardization, vital health information sharing, human capacity building, access and quality of healthcare delivery, and interoperability of systems. Also, policies must consider the eHealth processes by identification, prioritization, feasibility calculation, and evaluation of any action in advance.

### Step 3- develop guidelines/roadmap/framework for implementation

Based on the previous steps, considering the policy design and readiness outcome, and taking into account the local context, an implementation plan with proper guidelines or roadmaps should be developed based on its suitability for its targeted context.

### Step 4- evaluation and adaptation

There should be a continuous cycle of adaptation, evaluation, and refinement of strategies and policies to make an agile, flexible, and effective roadmap. This way, it is possible to contextualize the eHealth policy framework based on the short-term and long-term provisions or unpredicted incoming incidents.

## Conclusion

Despite huge investments by national governments to develop and implement eHealth solutions, little is known about how those investments are creating an impact at the local level in improving the quality of care and reducing the cost of healthcare delivery. As Scott & Mars 20,125 [9] clearly indicated, the lack of appropriate and context-based eHealth strategy is a barrier to the effective development and implementation of sustainable eHealth solutions in many developing countries. In recent years, there is a growing interest by international organizations (like WHO) and national governments in the importance and relevance of eHealth strategy and policy frameworks to reap the fruit of investments made in eHealth solutions. But, still, the literature lacks clear guidance and frameworks to inform countries and other stakeholders why and how to develop their own complementary but locally relevant eHealth strategy [9].

In this study, we conducted a review of the literature to explore the issues and factors that frame the implementation of eHealth strategy and policy frameworks in low and Lower Middle-income Countries. Our results demonstrate that there is a growing interest by government and non-government actors to spend on eHealth systems and to develop national policy frameworks. Governments and policymakers are also getting a good understanding of the great potential of eHealth to deliver cost-effective and quality healthcare both in rich and poor countries. However, our literature review revealed the often problematic and unsuccessful attempts to implement eHealth policy frameworks in the context of Low and Lower Middle-Income countries (LLMICs). As such, our review revealed the fact that a mixture of local/international approaches, principles, and standards are required to be able to implement an effective national eHealth strategy supported by national legislation and followed by strengthening policies in a framework that priorities are well appointed [24; Khumalo, 2017; Mars & Scott, 2010; 9; WHO, 2008].

Reviewing the literature to explore appropriate eHealth implementation frameworks for LLMICs, revealed the fact that a mixture of local/international approaches, principles, and standards are required to be able to implement an effective national eHealth strategy supported by national legislation and followed by strengthening policies in a framework that priorities are well appointed [8, 9, 24–26].

Several studies indicate one of the factors for slow progress in the adoption of eHealth in LLMICs is lack of proper policy frameworks to guide the development and implementation process. Our review also shows that in developing and implementing eHealth policy frameworks in LLMICs, it is crucial to approach it as a process with multiple issues and complexities to be addressed at each stage of the process. For example, before developing the policy framework, countries and organizations should explore issues related to organizational readiness, the availability of a competent labor force to implement the policy framework, and the suitability of existing legal and policy frameworks to implement the new policy framework. Similarly, while developing the policy framework our findings revealed the importance of considering international, national, regional, local, and institutional situations, as well as geographical, economic, cultural, infrastructural, and resource-related issues. During the implementation of the framework, the importance of developing guidelines (roadmaps) that are suitable to the local context was emphasized. The need for continuous post-implementation adaptation, evaluation, and refinement of strategies and policies has also been suggested to make an agile, flexible, and effective roadmap.

Moreover, since it was neglected in the literature and as it was clarified by the lack of correlations between the documents when discussing user-centeredness, engagement, interoperability, and standards, we suggest that there should be an important focus on these aspects if any framework is ever planned to be designed for developing countries. The movement toward patient-centricity and interoperability is inevitable and the necessity of standardization for interoperability, security, confidentiality, accessibility, availability, and storage of data is clearly noted by both experts and scholars [24, 25, 39, 41]. The limitation of this study is the lack of a proper amount of practically implemented eHealth policy framework cases published in the literature for the review.

To be able to overcome challenges and design an effective framework covering all these issues, the 4 steps proposed by this study are suggested for the decision-makers to better evaluate the situation, provision the needs, design and implement the strategies/policies/frameworks, and evaluate, develop, and refine it in a continuous cycle.

## Data Availability

The datasets used and/or analyzed during the current study are available from the corresponding author on reasonable request.

## Abbreviations

LLMICs: Low and Lower-Middle Income Countries
PRIMSA: Preferred Reporting Items for Systematic Reviews and Meta-Analyses
EHRs: Electronic Health Records
EMRs: Electronic Medical Records
WHO: World Health Organization
ICT: Information and Communication Technology
HMIS: Health Management Information System
UMICs: Upper-Middle Income Countries
TAPIC: Transparency Accountability Participation Integration Capacity
LSA: Latent Semantic Analysis
UX: User Experience
EU: European Union
NGO: Non-Governmental Organizations
IoMT: Internet of Medical Things
HR: Human Resources
